# Editorial to Radiation in Multimodal Tumor Immune Therapies—Mechanisms and Application

**DOI:** 10.3390/ijms22147648

**Published:** 2021-07-17

**Authors:** Benjamin Frey, Udo S. Gaipl

**Affiliations:** 1 Translational Radiobiology, Department of Radiation Oncology, Universitätsklinikum Erlangen, 91054 Erlangen, Germany; 2 Comprehensive Cancer Center Erlangen-EMN (CCC ER-EMN), 91054 Erlangen, Germany

The understanding and the application of radiation-induced immune modulation has become more and more relevant in the therapy of malignant, but also benign diseases. The review of Maier et al. [1] clarifies that even exposure to very low doses of radiation affects the immune system and could reduce pain in patients with chronic inflammatory and degenerative diseases. However, we are just beginning to understand the osteoimmunological modes of action of radon as a radioactive noble gas. Nevertheless, one can learn a lot, since the consequences of radon exposure depend on key factors such as intake and distribution way, chronic versus acute exposure, dose, basal inflammatory state, and radiation type, just to mention the most prominent examples. Kumari and colleagues [2] summarize the properties and application of different types of radiotherapy for cancer diseases in detail. A key general mechanism how radiation-induced DNA damage triggers immune signaling by DNA fragments being released in the cytoplasm of the cells is outlined. Again, it is stressed that radiation of different types (X-rays, protons, and carbon ions) has common and different properties and can induce DNA damage, trigger stress responses and provoke or suppress immunity. This knowledge together with the dynamics of the responses is of great importance for a proper design of multimodal cancer tumor therapies that include radiotherapy and immune therapies. Further, local effects are different from systemic, abscopal ones due to the different micro-environment and modulation of it by just local application of radiation. The local killing of tumor cells can be further improved by cold physical plasma that, like radiotherapy, induces reactive oxygen species that are, however, even more diverse. The preclinical work of Pasqual-Melo et al. [3] with the B16F10 melanoma model gives strong hints that the gas plasma treatment of tumors sensitizes the cells to subsequent radiotherapy rather than the other way around. This indicates a big challenge to the design of multimodal tumor therapies that should strongly consider not only the dynamics of radiation-induced local and systemic effects, but also the most beneficial sequence of the single applications. Wang et al. [4] illustrate this by another example with the biocompatible immunostimulant *N*-dihydrogalactochitosan (GC). Importantly, GC preferably increased the radiosensitivity of 4T1 breast cancer cells metastasized in the liver rather than that of the parental cells. This highlights that the micro-environment affects the radiosensitising effects of anti-cancer drugs including immune modulators. In this context, the vascular condition has to be considered, too. Horsman and colleagues [5] demonstrate in CDF1 mice bearing C3H mammary carcinomas that that vascular disrupting agents can make non-immunogenic tumors immunogenic. Punnanitinont et al. [6] point out that radiation has no predictable effect on expression of tumor antigens but rather it heightens expression of proteins involved in processing and presentation of antigens. The papers in this Special Issue clarify the mechanisms by which radiation contributes to the efficacy of multimodal therapies. They also indicate that while recent years have brought impressive advances in combination of radiotherapy with immune therapy, important opportunities for further insight and progress remain [7]. Let us go deeper from the tip of the iceberg.
